# A Comparative Analysis of Apical Rocking and Septal Flash: Two Views of the Same Systole?

**DOI:** 10.3390/jcm13113109

**Published:** 2024-05-25

**Authors:** Alexandra-Iulia Lazăr-Höcher, Dragoș Cozma, Liviu Cirin, Andreea Cozgarea, Adelina-Andreea Faur-Grigori, Rafael Catană, Dănuț George Tudose, Georgică Târtea, Simina Crișan, Dan Gaiță, Constantin-Tudor Luca, Cristina Văcărescu

**Affiliations:** 1 Doctoral School, “Victor Babes” University of Medicine and Pharmacy, 300041 Timisoara, Romania; alexandra.hocher@umft.ro (A.-I.L.-H.); liviu.cirin@umft.ro (L.C.); andreea.cozgarea@umft.ro (A.C.); 2 Institute of Cardiovascular Diseases Timisoara, 13A Gheorghe Adam Street, 300310 Timisoara, Romania; andreeaadelinafaur@yahoo.com (A.-A.F.-G.); simina.crisan@umft.ro (S.C.); dan.gaita@umft.ro (D.G.); constantin.luca@umft.ro (C.-T.L.); cristina.vacarescu@umft.ro (C.V.); 3 Department of Cardiology, “Victor Babes” University of Medicine and Pharmacy, 2 Eftimie Murgu Square, 300041 Timisoara, Romania; catanarafael02@gmail.com; 4 Research Center of the Institute of Cardiovascular Diseases Timisoara, 13A Gheorghe Adam Street, 300310 Timisoara, Romania; 5 County Clinical Emergency Hospital of Sibiu, 550245 Sibiu, Romania; 6 Institute of Cardiovascular Diseases C.C. Iliescu, Fundeni Clinical Institute, 258 Fundeni Street, 022328 Bucharest, Romania; georgedtudose@gmail.com; 7 Department of Physiology, University of Medicine and Pharmacy of Craiova, 200349 Craiova, Romania; georgica.tartea@umfcv.ro

**Keywords:** cardiac resynchronization, left ventricular dyssynchrony, septal flash, apical rocking, echocardiography

## Abstract

Heart failure (HF) is a complex medical condition characterized by both electrical and mechanical dyssynchrony. Both dyssynchrony mechanisms are intricately linked together, but the current guidelines for cardiac resynchronization therapy (CRT) rely only on the electrical dyssynchrony criteria, such as the QRS complex duration. This possible inconsistency may result in undertreating eligible individuals who could benefit from CRT due to their mechanical dyssynchrony, even if they fail to fulfill the electrical criteria. The main objective of this literature review is to provide a comprehensive analysis of the practical value of echocardiography for the assessment of left ventricular (LV) dyssynchrony using parameters such as septal flash and apical rocking, which have proven their relevance in patient selection for CRT. The secondary objectives aim to offer an overview of the relationship between septal flash and apical rocking, to emphasize the primary drawbacks and benefits of using echocardiography for evaluation of septal flash and apical rocking, and to offer insights into potential clinical applications and future research directions in this area. Conclusion: there is an opportunity to render resynchronization therapy more effective for every individual; septal flash and apical rocking could be a very useful and straightforward echocardiography resource.

## 1. Introduction

Heart failure (HF) is a clinical syndrome resulting from structural and functional impairment of ventricular filling or ejection of blood. The global incidence and prevalence rates of HF have reached epidemic proportions, affecting nearly 23 million people, and in the general European population the prevalence of symptomatic HF ranges between 0.4 and 0.6%, being similar to the prevalence in the United States [1]. The prevalence of HF rises exponentially with age, being ≥10% among people aged >70 years [2]. 

Within the population of patients with chronic HF, intraventricular conduction abnormalities play a substantial role in elevating the risk of morbidity and mortality. These abnormalities can lead to impaired systolic function, reduced left ventricular contractility, prolonged mitral regurgitation, and abnormal septal wall motion, affecting approximately one-third of patients with heart failure and reduced ejection fraction (HFrEF) [3,4,5,6,7,8,9]. It is noteworthy that the prevalence of secondary mitral regurgitation (SMR) in heart failure patients reaches 50% for ischemic and 65% for nonischemic cardiomyopathy, while mild to moderate secondary mitral regurgitation occurs in 49% of patients with HF and severe secondary mitral regurgitation occurs in 24% of patients [10,11]. The presence and severity of SMR alone reveal substantial connections with both all-cause mortality and heart failure hospitalizations [11]. 

Cardiac resynchronization therapy (CRT) is a well-established treatment for heart failure patients with a severely reduced ejection fraction (LVEF < 35%) and a QRS duration exceeding 130 ms who remain symptomatic despite optimized medical therapy [12]. Extensive data from large-scale randomized studies consistently show that, in patients with a wide left bundle branch block (LBBB) conduction pattern and severe left ventricular (LV) systolic dysfunction, cardiac resynchronization therapy (CRT) leads to significant improvements in hard outcomes such as reduced mortality [13] and heart failure hospitalization rates [14], as well as improvement of exercise capacity, symptoms, and quality of life [15]. In addition, cardiac resynchronization therapy (CRT) can reduce secondary mitral regurgitation by enhancing the closing forces driven by ventricular resynchronization as well as by diminishing the tethering forces on the mitral apparatus [16]. 

In the last three decades, extensive research efforts have focused on identifying imaging-based parameters that can uncover the electromechanical factors influencing the effectiveness of cardiac resynchronization therapy (CRT) [17]. Echocardiographic assessment of ventricular or mechanical dyssynchrony before CRT, irrespective of left ventricular ejection fraction (LVEF) or QRS duration [18,19,20] may enhance patient selection and identify patients who may benefit from CRT [9,21]. These assessments, including apical rocking and septal flash, have shown promise in predicting favorable outcomes in CRT patients, offering an accurate and rapid bedside investigation to guide treatment decisions [22,23,24,25,26,27,28,29]. 

During echocardiography examination, the most frequently observed abnormal wall motions are represented by septal flash, septal rebound stretch, and apical rocking. While both “septal flash” (early inward bulge of the septum) and “septal rebound stretch” (outward extension after initial shortening) are linked to the left bundle branch block (LBBB), their underlying condition is different. The origin of septal flash is in the early activation of the right heart chamber pushing on the unopposed septum, whereas rebound stretch is caused by late activation of the left ventricle after early septal contraction [30,31]. The prevalence of septal flash in LBBB patients exhibits a wide range, typically between 45% and 63%, with variations attributed to the specific population under study and the strictness of the criteria used to define LBBB [28,32]. 

Septal flash (originally termed “septal beak” in 1974 by Dillon et al.), when observed through M mode echocardiography in patients with left bundle branch block (LBBB) [33], serves as a prominent marker of intraventricular dyssynchrony.

The term “septal flash” gained specificity in 2008 when Parsai et al. linked it to an indicator of asynchrony resulting from electrical abnormalities [22], defining it as an anomalous, rapid leftward motion of the interventricular septum during the isovolumetric ventricular contraction phase, which occurs before the opening of the aortic valve [34,35]. This phenomenon is primarily driven by an electrical disorder, as the left bundle branch block leads to the left ventricle’s electrical activation via the right bundle branch, causing a depolarization process from right to left. This results in delayed contraction in the posterolateral walls due to slower propagation velocity through the myocardial fibers with distinct electrical properties compared to the specialized Purkinje system [30]. The septal strain pattern in the left bundle branch block (LBBB) is notable for its distinct features, which involve pre-ejection shortening followed by abrupt septal stretch during ejection [36,37].

The presence of a significant septal flash is frequently associated with a U-shaped activation pattern on electrical mapping and predicts a more favorable response to CRT intervention compared to patients with no or limited septal flash [28,38]. 

Moreover, in the context of left bundle branch block (LBBB), the septum, comprising approximately one-third of the left ventricle’s mass, loses its contractile effectiveness, leading to a dyssynchronous contraction that impairs the heart’s pumping function and results in the entire ejection fraction workload being carried by the posterior and lateral walls [39,40]. In cases of LBBB with a posterior lateral scar or myocardial ischemia, septal flash is notably absent, as these conditions do not exert the necessary forces to stretch the septum, potentially leading to a “pseudonormal” appearance initially, which can ultimately induce left ventricular remodeling [5]. This remodeling often manifests as hypertrophy of the left ventricular lateral wall and thinning of the septum [34], potentially driven by altered activation and stretch patterns in opposite LV walls that induce molecular and cellular changes [4], redistribute myocardial blood flow and oxygen uptake [41], and result in differences in septal-to-lateral wall thickness [42,43].

**Figure 1:**
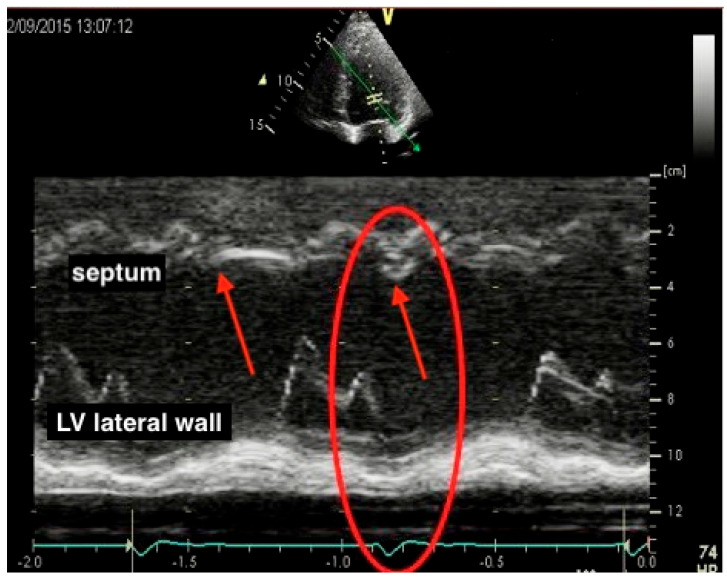
Septal flash on M-mode imaging.

An apical four−chamber view M−mode of the LV (Figure 1) is shown from a patient with typical LBBB, demonstrating septal flash (red arrows) and the dyssynchrony of contractility between the interventricular septum and posterolateral wall (red circle). The septum has an initial short inward motion before the ejection phase, which is followed by the stretching of the left ventricular lateral wall. 

Jansen et al. initially identified apical shuffle in 2007 as an unusual systolic septal-to-lateral movement of the left ventricle, yet they did not explore the underlying physiological mechanisms that cause this phenomenon, despite its predictive power for left ventricular reverse remodeling (sensitivity 90% and specificity 70%) [44]. Nowadays, the “apical shuffle” is known as apical rocking, and it is defined as abnormal lateral rocking of the ventricular apex caused by an intraventricular functional dyssynchrony. This dyssynchrony can be attributed to intraventricular conduction delays (e.g., LBBB), regional damage (e.g., myocardial scar), or a combination interplay of both [25,42,45,46]. 

Recently, the pathophysiological mechanism of apical rocking, characterized by brief early septal motion at the apex and a predominant lateral motion during ejection, has been elucidated in two distinct publications [26,47]. This phenomenon is characterized by a distinct right-to-left motion of the apical myocardium in the left ventricle (LV), occurring perpendicular to the LV’s long axis [47,48]. The mechanism of apical rocking involves a contraction that causes the septum to temporarily move inward, pulling the apex closer towards it. Following that, the septum stretches as the apex is pulled laterally by the delayed activation of the lateral wall during the ejection phase [49]. This gradual process allows the ventricle to build up the necessary force to eventually open the aortic valve and efficiently pump blood out. The extent of “apical rocking” in dilated cardiomyopathy directly relates to the size of the left ventricle (left ventricular end-diastolic volume), and the QRS duration does not influence the magnitude of the movement [50].

Even though each of these easily recognizable and accessible parameters indicates an atypical myocardial motion associated with the left bundle branch block (LBBB), our comprehension of the precise physiological mechanisms that underlie septal flash and apical rocking is restricted, regardless of their efficacy in the patient selection process for CRT. This literature review aims to provide a comprehensive analysis of the practical value of echocardiography for the assessment of left ventricular (LV) dyssynchrony using parameters such as septal flash and apical rocking, which have proven their relevance in patient selection for CRT, the relationship between septal flash and apical rocking, emphasize the primary drawbacks and benefits of using echocardiography for evaluation of septal flash and apical rocking, and offer insights into potential clinical applications and future research directions in this area.

**Figure 2:**
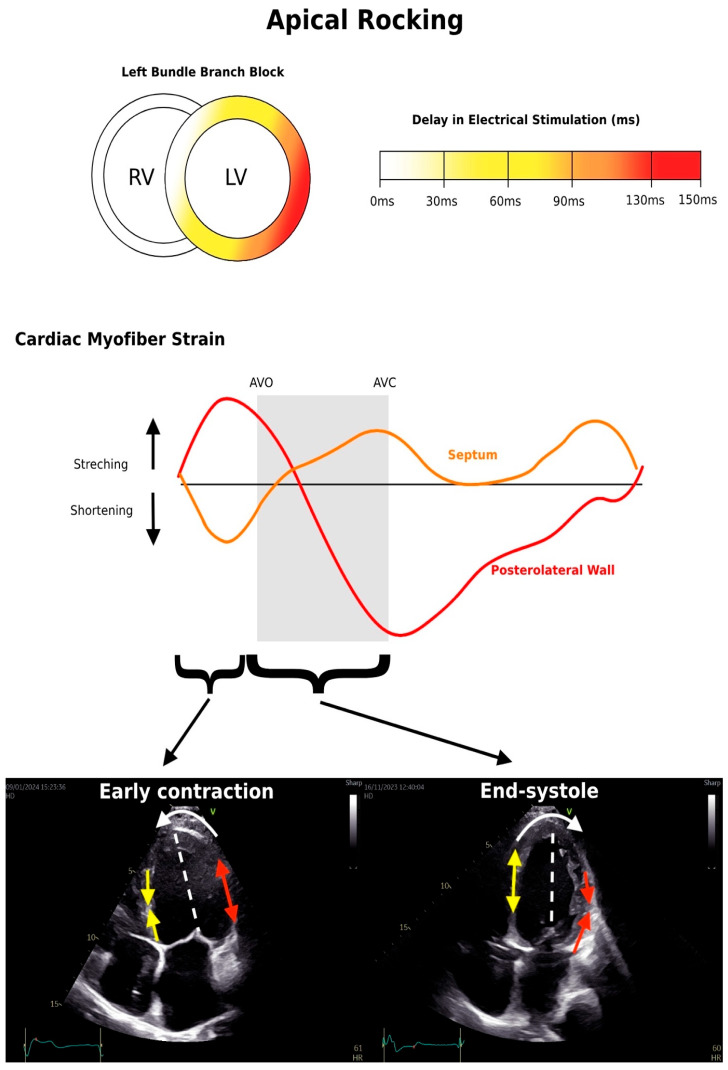
Computer Simulations of Cardiac Myofiber Strain in LBBB patients and two-dimensional echocardiography of apical four-chamber view illustrating the apical rocking phenomenon.

It is illustrated by the early septal contraction before aortic valve opening (AVO) associated with the pre-stretch and apical movement of the posterolateral wall towards the septum. At the end of the systolic phase, the apex undergoes rightward rotation due to the lateral wall contraction in addition to the septal stretching. The dashed line shows the longitudinal axis of the left ventricle (Figure 2).

## 2. Materials and Methods

The selection of studies for this review followed the PRISMA (Preferred Reporting Items for Systematic Reviews and Meta-Analyses) guidelines. This review was conducted by reviewing bibliographic searches on databases such as the PubMed database, Google Scholar, and Scopus. Both manual searches and the use of MeSH terms on PubMed were employed to identify articles on septal flash and apical rocking published within no date restriction. The selection of the most relevant articles involved assessing their titles, the information provided in their abstracts, and a brief overview of the complete manuscript. The eligibility criteria was based on studies that evaluated septal flash and/or apical rocking. There was no restriction based on how septal flash and/or apical rocking was defined, with all imaging modalities being included. Articles not written in English, publications with only abstracts available, and duplicate entries were excluded from consideration.

In October 2023, the search and selection process was carried out by two experienced cardiologists with expertise in interventional arrhythmology and echocardiography. Initially, we manually scoured articles using the specific keywords “ventricular dyssynchrony” and “septal flash” and “apical rocking”. Additionally, using the MeSH term option available in PubMed, we conducted another search with the following terms: ((“Cardiac Resynchronization Therapy” [Mesh]) AND “Echocardiography”[Mesh]).

**Figure 3:**
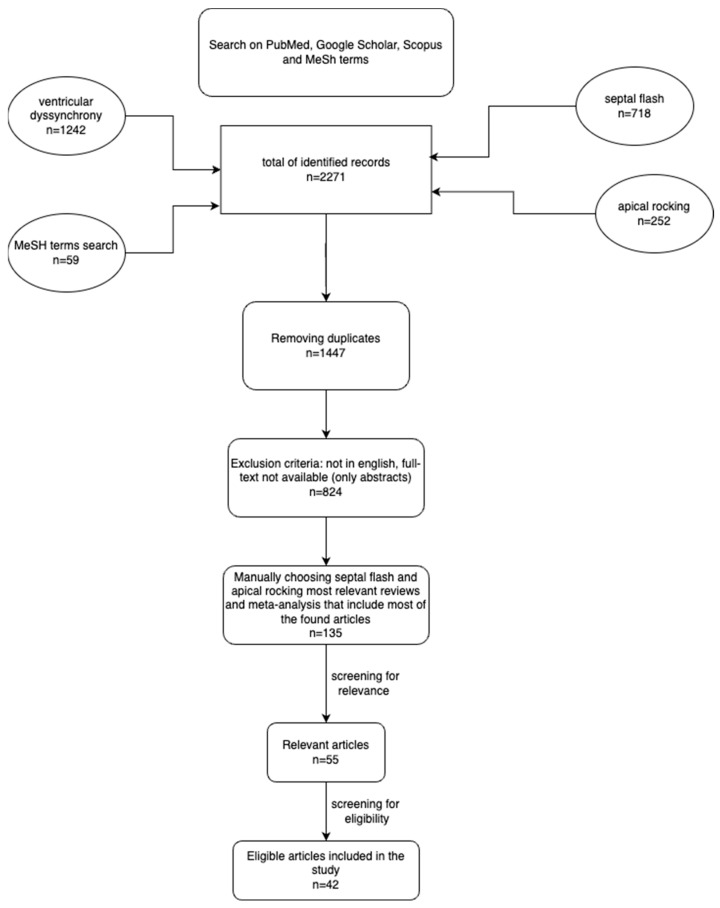
PRISMA chart of the article selection.

The PRISMA diagram below illustrates the search strategy employed along with the filters that were applied (Figure 3). The diagram was made using the draw.io application (UK).

To enhance the organization and planning of the review, all chosen articles were integrated into a Microsoft Excel (version 2021) table. This table included columns for the article’s title, authors, utilized imaging method, year of publication, journal, publication type, and keywords.

We deliberated on the critical data and findings, with a particular emphasis on the correlation (Table 1) and the difference (Table 2) between septal flash and apical rocking. We categorized the articles in the following manner:

1. The strong inter-relationship between septal flash and apical rocking—19 selected articles [studied included in quantitative synthesis (*n* = 16); studied included in qualitative synthesis (*n* = 3)]; some of the most relevant, of date articles regarding the same underlying pathophysiology similarities and differences.
2. The importance of echocardiography in ventricular dyssynchrony management—23 selected articles [studied included in quantitative synthesis (*n* = 19); studied included in qualitative synthesis (*n* = 4)]; some of the most relevant articles regarding the role and limitation of the echocardiographic techniques were selected and compared with one another.

## 3. Results

### 3.1. The Relationship between Septal Flash and Apical Rocking

Septal flash and apical rocking are related features of the typical left bundle branch block (LBBB) contraction pattern, indicating a shared underlying pathophysiology. They often coexist in the same patients, although their extent may vary.

Stankovic et al. determined that the left bundle branch block (LBBB) causes early septal contraction, seen as “septal flash”, occurring before the aortic valve opens [28]. This is a quick inward and outward motion of the septum. During this septal flash, the left ventricular (LV) apex moves toward the septum, initiating the first part of the apical rocking motion. Subsequently, a delayed and more pronounced posterolateral contraction leads to stretching of the septum and movement of the LV apex in the opposite direction, completing the second phase of the apical rocking, as can be observed by experienced observers in an echocardiographic four-chamber view of a heart with dyssynchrony [22,26,30,49]. The main distinction between them lies in the timing of the cardiac cycle. Septal flash takes place during the shorter isovolumic ventricular contraction phase, whereas apical rocking occurs during ejection, which has a longer duration. The presence of septal flash and apical rocking corresponds with research that uses quantitative assessments of systolic stretch by strain imaging [37]. 

However, it is worth noting that they can occasionally be observed together or separately, with variations in their intensity [28]. There are some several conditions that can contribute to the diminished intensity or even the complete dispersal of these phenomena, summarizing that [30,36,51]: the passive phase of the septal flash can be impaired by contractility dysfunction or high filling pressures of the right ventricle; the active phase of the septal flash can be diminished by a delayed conduction through the right bundle branch or the presence of a septal scar; a scarred posterolateral wall will reduce the ability of the septum to stretch during the second phase of apical rocking; slow RBB conduction that is disguised inside the LBBB, resulting in slow RV contraction and septal activation (this would suggest that SF might occur only when RBB conduction is intact); a septal fascicle could bypass the slow transseptal conduction; difficulties in the alignment of the ultrasound fascicle beam with the latero-lateral movement of the septal flash or a low frame rate can impair the visualization of the septal flash [52]; the site of the left bundle branch block is important because, the more proximal the block is, the more typical septal flash is [30,36,51,53,54].

Alternatively, isolated mechanical asynchrony can imitate the genuine septal flash induced by electrical conduction irregularities. Moreover, elevated right ventricular pressures or a scarred septum can trigger a right-to-left systolic motion that may resemble septal flash. In these instances, the critical distinguishing characteristic is that the systolic motion persists longer than in the case of a genuine septal flash [20]. 

### 3.2. Echocardiography

Given its widespread availability, feasibility, and capacity to assess regional myocardial function with excellent temporal and spatial resolution, echocardiography emerges as an ideal imaging tool for the selection of patients suitable for cardiac resynchronization therapy (CRT) [49].

Early investigations into left ventricular mechanical dyssynchrony using echocardiography primarily concentrated on quantifying the delay in the contraction of opposing ventricular walls. In a small-scale study involving 24 heart failure patients, Pitzalis et al. identified that a septal-to-posterior motion delay (SPWMD) of ≥130 milliseconds could serve as a predictor of left ventricular reverse remodeling in patients without a history of anterior or septal infarction [55,56]. Later investigations explored the use of tissue Doppler imaging and speckle-tracking echocardiography to assess LV dyssynchrony. These techniques were employed to measure opposing wall delays or analyze variations in peak systolic velocities among different myocardial regions [13,18,57].

Another technique proposed to assess left ventricular dyssynchrony involves analyzing the unique contraction pattern associated with LBBB and comparing the contractility of opposing walls to identify septal flash (SF) [22] and/or apical rocking (ApR) [25]. 

Given that there is no universally preferred method for assessing these parameters, various techniques have been suggested. Septal flash is conventionally evaluated using M-mode in the parasternal long-axis view, as well as 2D and tissue Doppler imaging in either the short or long parasternal long-axis view [22,58]. The M-mode echocardiography can be applied from the parasternal views to ascertain the radial function of the interventricular septum, and the evaluation of the longitudinal displacement of the interventricular septum can be determined from the apical views using TDI-derived speckle tracking [7,59]. However, relying on longitudinal strain for assessing the accurate pattern of septal radial movement is not precise [38]. Assessing radial strain is a more intricate task, while parameters derived from longitudinal strain, such as septal rebound stretch and systolic stretch index [60,61], are more readily available, reproducible, and commonly employed in the selection of CRT patients. Marechaux et al. described three echocardiographic patterns of septal flash by applying the longitudinal strain speckle tracking specifically, as follows: pattern 1—a double-peaked systolic shortening; pattern 2—an early pre-ejection shortening peak followed by a prominent systolic stretch; pattern 3—a pseudo-normal shortening with a late systolic shortening peak, no or minimal pre-ejection septal lengthening, and less pronounced end-systolic stretch. This study concluded that the first two patterns of septal flash were strongly linked to a positive response to CRT, while the third pattern was more likely to lead to a negative response. The third pattern was also associated with a high level of scarring in the heart muscle and a more advanced stage of heart failure [62].

Voight et al. devised an algorithm for quantifying apical rocking through the measurement of apical transverse motion (ATM), which combines the longitudinal myocardial data from the interventricular septum and the lateral wall of the left ventricle. Their findings indicated that apical transverse motion serves as a valuable parameter for enhancing the identification of patients who stand to benefit from CRT therapy [25].

Nevertheless, echocardiography has significant limitations (Table 3). This approach relies on complex and time-consuming algorithms for assessing different cardiac cycles, and myocardial peak velocities can be greatly affected by factors like the filling condition and the passive motion of the analyzed wall segment. Furthermore, the expertise of the echocardiographer is essential for the accurate diagnosis of septal flash and apical rocking in candidates for cardiac resynchronization therapy (CRT). In a small, single-center study, Mada et al. demonstrated that semi-automatic detection of these particular contraction patterns using speckle tracking echocardiography was superior to inexperienced echo-readers for recognizing SF/ApR [63].

## 4. Discussion

Echocardiography plays a pivotal role in the selection of patients undergoing cardiac resynchronization therapy (CRT) due to its broad spectrum of possibilities. Septal flash and apical rocking are practical indicators in predicting the response to cardiac resynchronization therapy (CRT), resulting in improved clinical outcomes for patients.

Cardiac resynchronization therapy (CRT) plays an essential role in treating heart failure patients with prolonged QRS duration and low LVEF [12]. International guidelines make a Class IA recommendation for CRT device implantation in symptomatic patients in sinus rhythm with an LV ejection fraction (LVEF) of ≤35%, LBBB and QRS duration (QRSd) of ≥150 ms, despite optimal medical therapy and a Class IIaB for symptomatic patients with heart failure in sinus rhythm with an LV ejection fraction (LVEF) of ≤35%, LBBB and QRS duration (QRSd) 130–149 ms, despite optimal medical therapy for the reduction in mortality and morbidity [12]. Despite the coexistence of electrical and mechanical dyssynchrony, the CRT guideline recommendations rely on electrical dyssynchrony criteria, due to the amount of research data using QRS duration as an enrollment criterion and findings from studies like the Predictors of Response to CRT (PROSPECT) study [13].

However, certain research data proved that septal flash is a relevant echocardiographic finding in over half of heart failure (HF) patients receiving cardiac resynchronization therapy (CRT), and it serves as a valuable predictor of favorable left ventricular reverse remodeling following CRT implantation, as evidenced by Doltra et al., who reported an 80.2% echocardiographic response rate with reductions in LV end-systolic volumes and LVEF, along with CRT-induced correction of septal flash in 93% of patients [64]. Gabrielli et al. determined septal flash as the only independent predictor of significant left ventricular reverse remodeling [29], Gąsior et al. observed that septal flash at baseline correlated with notable enhancements in LVEF, LV end-systolic volumes, and diastolic volumes [65], and Parsai et al. reported that 88% of cases displayed the resolution of septal flash after CRT implantation, along with improvements in LV volumes, although no significant overall LVEF improvement was noted [22]. In a comprehensive, multi-center observational study known as PREDICT-CRT, a quantitative measurement of apical rocking was compared with visual assessment and showed similar accuracy in predicting the response to CRT. In addition, Stankovic et al. demonstrated that a certain pattern of LV mechanical dyssynchrony, characterized by apical rocking and septal flash, is associated with enhanced long-term survival after cardiac resynchronization therapy (CRT) [28]. Another study conducted by Parsai et al. proved that low-dose dobutamine stress echocardiography (DSE) represents a valuable approach for revealing septal flash in individuals with a viable septum, irrespective of the cause of their heart failure [23]. Low-dose DSE unveils or amplifies the extent of dyssynchronous motion induced by left bundle branch block (LBBB) and helps in the identification of patients who may experience reverse remodeling following cardiac resynchronization therapy (CRT). CRT responders exhibit a characteristic early septal contraction that pulls the apex toward the septum, aligning with other studies demonstrating this phenomenon as a brief bounce in the septum, known as septal flash [22]. The prominence of septal flash is an indicator of the inefficient work performed by the dyssynchronous left ventricle, and its disappearance following pacing is associated with the reversal of left ventricular remodeling, whereas its persistence after pacing is a sign of non-responsiveness to therapy [23]. Furthermore, Stankovic et al. concluded that the presence of septal flash and apical rocking has shown its predictive value for CRT response in individuals with chronic right ventricular pacing who require an upgrade to cardiac resynchronization therapy (CRT) [66]. 

Although small cohort retrospective studies have shown positive outcomes related to the utility of echo-derived parameters in predicting CRT response, the extensive PROSPECT trial did not succeed in confirming their effectiveness for identifying appropriate CRT candidates [67]. These inconclusive outcomes are ascribed to the variability in the reproducibility of echo-derived dyssynchrony parameter analysis [21], as well as the inadequacy of opposite wall delay as the sole assessment tool, which fails to fully elucidate the complex pathophysiology underlying CRT response.

Even though randomized controlled trials (RCTs) have demonstrated significant benefits of cardiac resynchronization therapy (CRT) in HFrEF patients (Table 4), a subset exhibits non-response characterized by persistent symptoms, recurrent hospitalizations for heart failure, and lack of left ventricular reverse remodeling. Non-responsiveness to CRT can be attributed to a broad range of causes, including poor lead placement in the left ventricle, suboptimal atrioventricular (AV) and ventricular-ventricular (VV) timing, left ventricular scar, and progression of heart failure [68]. The excessive focus on the idea of “non-response” to cardiac resynchronization therapy (CRT) has led to a significant lack of use of the device, leading to up to two-thirds of indicated patients failing to obtain the implant [69]. The most recent attempt in the data area focuses on identifying patients who respond to the CRT with a particularly beneficial (super) response patients because of their significantly lower cumulative risk of heart failure, mortality, and need for defibrillator therapy for ventricular tachycardia or ventricular fibrillation. Based upon the results of previous research, studies have identified the following two potential predictors of a “super-response” to treatment: left bundle branch block (LBBB) and a smaller size of the left atrium [70,71]. The MADIT-CRT trial identified female gender, absence of prior myocardial infarction, QRS duration ≥ 150 ms, LBBB, BMI < 30 kg/m^2^, and reduced left atrial volume index as predictors of super-responsiveness to cardiac resynchronization therapy [72]. 

Based on the fact that these two parameters are distinctive characteristics of left bundle branch block (LBBB) contraction patterns, which indicate the presence of an asynchronous left ventricular (LV) contraction, the goal of cardiac resynchronization therapy (CRT) is to correct the dyssynchrony by coordinating the contraction of the ventricles. In consequence, a coordinated contraction will enhance the function of the left ventricle and will decrease the burden of the symptoms of heart failure. In addition, the relationship between septal flash and apical rocking and favorable outcomes in patients undergoing cardiac resynchronization therapy (CRT) may be related to their link with left ventricular reverse remodeling [29,67]. The literature data have shown that there is a link between the resolution of septal flash and the reduction in apical rocking post cardiac resynchronization therapy (CRT) implantation with an increase in left ventricular (LV) function, defined as decreased end-systolic volumes [22] and increased ejection fraction percentage [65]. In patients undergoing cardiac resynchronization therapy (CRT), the presence of septal flash and apical rocking may therefore function as indicators of CRT response and subsequent left ventricle (LV) reversal remodeling, resulting in enhanced clinical outcomes [28].

However, the particular mechanisms beneath the efficacy of cardiac resynchronization treatment (CRT) for individuals with heart failure and widened QRS remain unclear, emphasizing the need for a more comprehensive assessment of their pathophysiology because dyssynchrony might be probably the most important factor, but not the only factor, influencing the success of CRT in an individual patient [49]. This increased comprehension could lead to a more accurate and focused evaluation of patients for CRT, possibly outlining the wide range of responses noticed, from significant LV reverse remodeling in “CRT super-responders” [72] to more modest effects or even deterioration of LV function [73,74,75]. Alternative CRT pacing, such as fusion pacing CRT or LV only pacing, was associated with a high rate of super-responders [76,77]. Fusion pacing CRT is defined as optimised LV only pacing (with or without a back-up RV lead) creating electrical and mechanical fusion with the intrinsec QRS. Avoiding RV pacing may substantially increase the structural response rate by shortening LV activation time [78]. Moreover, different ”degrees” of LV only fusion, according to AV node variability and heart rate, seem to play an important role in the LV remodelling process, and post-implantation optimization by targeting electrical dyssynchrony can improve CRT response in non-responder patients [79,80]. In consequence, understanding all components of cardiac dyssynchrony is essential not only in patient selection, but also in choosing the best CRT pacing modality.

Comparing our results with those of previous studies reveals consistencies and discrepancies both in terms of results and clinical implications. Our findings mainly align with previous research in highlighting the importance of echocardiography in assessing cardiac function and identifying potential candidates for cardiac resynchronization therapy (CRT), but we also highlight the ongoing challenge of assessing septal flash and apical rocking despite their formerly established significance in improving patient selection. Compared to previous research that may have indicated specific criteria for parameters like septal flash and apical rocking [20,26,30], our results contribute to the consensus that no single echocardiographic approach has gained broad support in current clinical guidelines.

Furthermore, our review emphasizes the tendency to underestimate the strong correlation between septal flash and apical rocking. Although they often coexist in patients with left bundle branch block (LBBB) and left ventricle (LV) dyssynchrony, the inadequate application of both in the approach of these patients is neglected, and there is not enough data on the combined effectiveness of these parameters. Along with that, adopting an integrated strategy towards heart failure patients undergoing cardiac resynchronization therapy (CRT) increases the potential to significantly influence the prognosis of the group of patients now referred to as non-responders.

This review contributes to the existing literature data by providing a comprehensive assessment of septal flash and apical rocking, their close relationship, and their potential relevance. Even more, by addressing gaps in the understanding of echocardiographic analysis, our research summarizes the current evidence on septal flash and apical rocking, emphasizing that echocardiography is the most accessible, readily available imaging modality that can improve patient selection.

This review has certain limitations due to the lack of studies examining septal flash and apical rocking. Our work is restricted by the absence of established criteria for measuring septal flash and apical rocking, leading to inconsistencies during echocardiography. More research is needed to fill in the lack of data in this area. Specifically, studies should focus on quality of life measurements, cardiac event rates (such as recurrent hospitalization with heart failure), and mortality rates to thoroughly evaluate the association of septal flash and apical rocking and their potential impact on mortality.

## 5. Future Direction

The future perspectives of the clinical application of apical rocking and septal flash for patients undergoing cardiac resynchronization therapy (CRT) display potential in enhancing tailored therapeutic approaches and optimizing outcomes for patients. Additionally, septal flash and apical rocking may be useful parameters for tracing the evolution of CRT response [28]. Through regular assessment of these echocardiographic parameters following implantation, clinicians can continue to monitor changes in ventricular dyssynchrony and assess the effectiveness of the therapy. By applying this dynamic monitoring strategy, non-responders can be detected earlier, which allows for prompt adjustments to be implemented to optimize patient outcomes. Further research into the possible integration of apical rocking and septal flash into risk stratification models for heart failure patients may be appropriate as our understanding of their prognostic significance advances. By using clinical and demographic data with these echocardiographic parameters, it may be possible to improve risk prediction tools for the aim of selecting patients who are most prone to adverse outcomes and providing guidance for targeted interventions, such as cardiac resynchronization therapy (CRT) [18,20,26,48]. The clinical application of apical rocking and septal flash in CRT patients should benefit from validation in real-world clinical practice settings. Prospective studies should focus on patient-centered-outcome research to evaluate the effects of apical rocking and septal flash on quality of life, functional status, and healthcare use in patients who have undergone cardiac resynchronization therapy (CRT).

## 6. Conclusions

In conclusion, this review provides a comprehensive analysis of the practical effectiveness of septal flash and apical rocking which are valuable echocardiographic parameters to assess the left ventricle (LV) dyssynchrony and to guide the patient selection for cardiac resynchronization therapy (CRT). Even if both parameters are predictive and assist in enhancing potential cardiac resynchronization therapy (CRT) responders, their dyssynchrony causes, assessment methods, and clinical implications differ. However, their combined evaluation improves the assessment of left ventricle (LV) dyssynchrony and the cardiac resynchronization therapy (CRT) optimization for better outcomes.

## Figures and Tables

**Table 1:** Similarities of septal flash and apical rocking.

Feature	Septal Flash	Apical Rocking
Type of abnormality	Wall motion abnormality	Wall motion abnormality
Pathophysiology	Early activation of the right ventricle exerts pressure on the septum as a consequence of left bundle branch block (LBBB) [20,30]	Early septal contraction pulls the apex inwards, while delayed lateral wall contraction pulls the apex outwards [26,28]
Associated with	The uncoordinated contraction of the left ventricle [20,22,28,30,36,51]	The uncoordinated contraction of the left ventricle [26,28]
Observed with	Echocardiography	Echocardiography

**Table 2:** Differences of septal flash and apical rocking.

Feature	Septal Flash	Apical Rocking
Type of abnormality	Paradoxical interventricular septum movement toward left ventricle during systole [20,30,36]	Brief early septal motion at the apex and a predominant lateral motion during ejection [26,47]
Region	Involves the interventricular septum [20,22,30,36,51]	Primarily affects the left ventricular apex [28]
Timing in the cardiac cycle	Earlier—During isovolumic contraction phase [20,30]	Later—During the ejection phase [26,28]
Clinical Significance	Associated with left bundle branch block (LBBB) and other conduction abnormalities [20,30,49]	Typically seen in patients with left ventricular dysfunction or ischemic heart disease [26]

**Table 3:** Advantages, disadvantages, strengths, and limitations of echocardiography in the assessment of septal flash and apical rocking.

Parameter	Septal Flash	Apical Rocking
Advantages	A variety of echocardiographic techniques, including M-mode, 2D, and tissue Doppler imaging [18,55,60,62] Various septal flash echocardiographic patterns have been identified, some of which are strongly associated with a favorable response to CRT [38]	Enhances identification of patients who may benefit from CRT [25] Quantified through apical transverse motion (ATM) [25]
Disadvantages	Myocardial peak velocities can be influenced by a multitude of variables, such as passive motion and filling condition [7] Expertise of echocardiographer is essential [63]	Assessment complexity and reliance on expertise of the operator [63]
Strengths	Potential predictive value for CRT response [7,22] Different septal flash patterns were identified [38,62]	An additional parameter is provided to improve the process of selecting patients for CRT Quantifiable through specific echocardiographic measurements [25]
Limitations	Operator dependency and complexity of interpretation [56,63] Semi-automatic detection may not completely replace expertise of an experienced Echocardiographer [63] Specific septal flash patterns might not be common to all Patients [38,62] Features such as the severity of heart failure and a history of myocardial fibrosis may impact the analysis	Relies on expertise of echocardiographer for accurate evaluation [63] Algorithmic quantification may still be influenced by various factors affecting myocardial motion such as temporal and regional function Inhomogeneities [25]

**Table 4:** Summary of the main cited studies providing an overview of the relationship between septal flash and apical rocking in the field of CRT.

Authors/Journal-Year/Ref	Population Study	Endpoints	Imaging Parameters	Results
C. Parsai et al., Eur. Heart J., 2009 [22]	161 pts 66 ± 10 years LVEF 24 ± 6% QRS width >120 ms	Reverse remodeling was defined as a reduction in LVESV ≥10%	SF	Presence of SF Se 64%, Sp 55%, PPV 81%, NPV 33% for the prediction of LV response remodeling
		Clinical response was defined as a reduction in NYHA Class ≥1		
C. Parsai et al., Eur. Heart J., 2008 [23]	52 pts 69 ± 2 years LVEF 24 ± 7% QRS width >120 ms	Reduction in LVESV ≥10%	SF	Presence of ApR and SF Se 84% and 79%, Sp 79% and Sp 74%, and accuracy of 82% and 77% for the prediction of LV response remodeling
		Clinical response was defined as a reduction in NYHA Class ≥1, increase in 6 min walk test (>10%), and fall in BNP (≥30%)		ApR [HR] 0.40, 95% confidence interval [Cl] 0.30–0.53, *p* < 0.0001 and SF (HR 0.45 [Cl 0.34–0.61], *p* < 0.001 is associated with lower all-cause mortality after CRT
M. Szulik et al., Eur. J. Echocardiogr., 2010 [26]	69 pts 63 ± 10 years	Reduction in LVESV > 15%	ApR	Presence of ApR resulted in an average Se = 89%, Sp = 75%, and accuracy for prediction of CRT response of 83%;
I. Stankovic et al., Eur. Heart J., 2014 [27]	58 pts 63 ± 10 years LVEF 26 ± 6% QRS width > 175 ± 25 ms	An increase >5% of LVEF during stress-echocardiography	ApR	AUC 0.88, 95% CI 0.77–0.99, *p* < 0.001 for the prediction of CRT response
I. Stankovic et al., Eur. Heart J.—Cardiovasc. Imaging, 2016 [28]	1060 pts 64 ± 11 years LVEF 27 ± 7% QRS width >170 ± 29 ms	Reduction in left LVESV ≥15% during the first year FU	SF ApR	Presence of ApR and SF Se 84% and 79%, Sp 79% and Sp 74%, and accuracy of 82% and 77% for the prediction of LV response remodeling
				ApR [HR] 0.40, 95% confidence interval [Cl] 0.30–0.53, *p* < 0.0001 and SF (HR 0.45 [Cl 0.34–0.61], *p* < 0.001 is associated with lower all-cause mortality after CRT
L. Gabrielli et al., Europace, 2014 [29]	94 pts 69 ± 8 years QRS width >166 ± 35 ms	Reduction in LVESV >15%	SF	Baseline SF predicts CRT response (OR 5.24; 95% Cl 1.95–14.11)
Z. Gąsior et al., Arch. Med. Sci., 2016 [65]	133 pts 63 ± 10 years LVEF 25 ± 6% QRS width >165 ± 25 ms	Evaluation of SF implication	SF	SF presence before CRT implantation is related to significant improvement in LV systolic and diastolic volumes and LVEF (*p* < 0.05)
		Evaluation of myocardial contractile reserve for 12 months follow up		
A. Ghani et al., Neth. Heart J., 2016 [48]	297 pts median age 68.7 years median LVEF 24 ± 8% median QRS width 160 ms	Determination of the independent association of LV ApR and CRT super-response	ApR	Super-responders had an absolute mean LVEF increase of 27% (22.05 ± 5.7 at baseline and 49.0% ± 7.5 at follow up) ApR was more common in super-responders (76%, *p* < 0.001)
A. Doltra et al., JACC Cardiovasc. Imaging, 2014 [67]	200 pts 67.37 ± 9.17 years LVEF 24 ± 6% QRS width >169.29 ± 30.38 ms	Reduction in LVESV ≥15%	SF	Baseline SF predicts CRT response in 93% of the population

Abbreviations: ApR—apical rocking, AUC—area under the curve, BNP—brain natriuretic peptide, CI—confidence interval, CRT—cardiac resynchronization therapy, FU—follow-up, LV—left ventricle, HR—hazard ration, LVEF—left ventricle ejection fraction, LVESV—left ventricle end-systolic volume, NPV—negative predictive value, NYHA—New York Heart Association functional class, OR—odds ration, PPV—positive predictive value, Se—sensibility, Sp—specificity, SF—septal flash.

## Data Availability

Data sharing is not applicable.
